# Molecular modelling approaches predicted 1,2,3-triazolyl ester of ketorolac (15K) to be a novel allosteric modulator of the oncogenic kinase PAK1

**DOI:** 10.1038/s41598-021-96817-3

**Published:** 2021-09-01

**Authors:** Md Shahinozzaman, Sinthyia Ahmed, Rashiduzzaman Emran, Shinkichi Tawata

**Affiliations:** 1 PAK Research Center, University of the Ryukyus, Okinawa, Japan; 2 Department of Bioscience and Biotechnology, Faculty of Agriculture, University of the Ryukyus, Senbaru 1, Nishihara-cho, Okinawa, 903-0213 Japan; 3 Division of Computer Aided Drug Design, The Red-Green Research Center, Dhaka, Bangladesh; 4 Bioscience and Bioinformatics Research Center (BBRC), 5/2, Shehora, Dhaka Road, Mymensingh, 2200 Bangladesh; 5 Department of Agricultural Extension (DAE), Khamarbari, Farmgate, Dhaka, 1215 Bangladesh; 6 Present Address: Department of Nutrition and Food Science, University of Maryland, College Park, MD 20742 USA

**Keywords:** Computational biology and bioinformatics, Drug discovery, Cheminformatics

## Abstract

P21-activated kinases (PAKs) are serine/threonine protein kinase which have six different isoforms (PAK1–6). Of those, PAK1 is overexpressed in many cancers and considered to be a major chemotherapeutic target. Most of the developed PAK1 inhibitor drugs work as pan-PAK inhibitors and show undesirable toxicity due to having untargeted kinase inhibition activities. Selective PAK1 inhibitors are therefore highly desired and oncogenic drug hunters are trying to develop allosteric PAK1 inhibitors. We previously synthesized 1,2,3-triazolyl ester of ketorolac (15K) through click chemistry technique, which exhibits significant anti-cancer effects via inhibiting PAK1. Based on the selective anticancer effects of 15K against PAK1-dependent cancer cells, we hypothesize that it may act as an allosteric PAK1 inhibitor. In this study, computational analysis was done with 15K to explore its quantum chemical and thermodynamic properties, molecular interactions and binding stability with PAK1, physicochemical properties, ADMET, bioactivities, and druglikeness features. Molecular docking analysis demonstrates 15K as a potent allosteric ligand that strongly binds to a novel allosteric site of PAK1 (binding energy ranges – 8.6 to – 9.2 kcal/mol) and does not target other PAK isoforms; even 15K shows better interactions than another synthesized PAK1 inhibitor. Molecular dynamics simulation clearly supports the stable binding properties of 15K with PAK1 crystal. Density functional theory-based calculations reveal that it can be an active drug with high softness and moderate polarity, and ADMET predictions categorize it as a non-toxic drug as evidenced by in vitro studies with brine shrimp and fibroblast cells. Structure–activity relationship clarifies the role of ester bond and triazol moiety of 15K in establishing novel allosteric interactions. Our results summarize that 15K selectively inhibits PAK1 as an allosteric inhibitor and in turn shows anticancer effects without toxicity.

## Introduction

P21-activated kinases (PAKs) are serine/threonine protein kinases which function as effectors of small GTPases Ras-related C3 botulinum toxin substrate (Rac) and cell division control protein 42 (Cdc42), and hence, they are also called Rac/Cdc42-activated kinases. They are the key cytoskeletal regulatory proteins having six different PAK isoforms in mammals and are categorized into two distinct groups based on their sequences and functions^1^. Group I comprises PAK1–3 which are activated by both Rac and Cdc42, and group II includes PAK4–6 which preferably bind to Cdc42 over Rac during their activation^2^. Through regulating cytoskeleton modelling, PAKs affect cell shape, motility, adhesion, cell proliferation and survival, being positioned at the intersection of several important cellular signaling pathways^2, 3^. They were reported to be overexpressed in many human cancers and generally exhibit a positive correlation with advanced tumor grade and decreased patient survival^4, 5^. Besides, most PAK isoforms have oncogenic signaling effects in cells, including the acquisition of growth signal autonomy, evasion of apoptosis, and the promotion of invasion and metastasis, when overexpressed or aberrantly activated^3^. Particularly, PAK1 and PAK4 are often upregulated in human tumors, and the tumor cells with upregulated PAK1 and PAK4 tend to become dependent on PAK signaling^3^. PAK1 is also responsible for developing many other human diseases/disorders including inflammation, hypertension, type II diabetes, neurofibromatosis, Alzheimer's disease, epilepsy, schizophrenia, depression, autism, obesity, and thereby shortening the lifespan^6^. PAK1-knockout *Caenorhabditis elegans* shows increased lifespan over the wild type one, whereas the absence of PAK4 in mice led to embryonic death and defects in neuronal development, clarifying that PAK4 is necessary for embryonic and neuronal growth^7^. However, in a previous study, PAK4 inhibitors were also reported to cause intestinal mucosa injury^8^. Thus, the oncogenic drug hunters have recently identified PAK1 to be an important and safe chemotherapeutic target as its inhibition does not cause toxicity to the normal cellular development and functions, which is usually caused by the traditional chemotherapeutic agents being also termed as microtubule poisons^6^. Nowadays, the world leading pharmaceutical companies maintain kinase programs to develop kinase inhibitors including PAK1 inhibitors especially as chemotherapeutics, and the developed small molecule drugs yet function as type I or type II inhibitors that typically target the highly conserved ATP-binding pocket of kinases. Due to having similarities in ATP-binding pockets of different kinases, specificity issues are common for active inhibitors, and cross-reactivity causes unwanted toxicities^5^. Therefore, targeting allosteric pockets of kinases outside the conversed ATP pocket has been proposed nowadays as a promising alternative to overcome current barriers of kinase inhibitors, including poor selectivity and toxicity, and the inhibitors which could bind to the allosteric pockets are two types—type III and type IV inhibitors. Selective PAK1 inhibitors are now highly desired since pan-PAK inhibitors show toxic effects through their promiscuous activities. To develop selective PAK1 blockers through adopting two different approaches—firstly using herbal drugs from the fruits, vegetables, and medicinal plants and secondly through chemical modification of the existing bioactive compounds/drugs—is the major goal of our group, and recently, a highly potent PAK1 blocker, called 15K (1,2,3-triazolyl ester of ketorolac), has been developed from a non-steroidal anti-inflammatory drug (NSAID), ketorolac, using click chemistry technique^9^. 1,2,3-Triazole-based heterocycles have been well exploited for generating medicinal scaffolds as anti-HIV, anticancer, antibacterial, anti-inflammatory, anti-parasitic, and antithrombotic drugs. They have also been reported as various enzyme inhibitors such as histone deacetylase, phosphodiesterase-4, alkaline phosphatase, cysteine protease, and acetylcholinesterase, and many 1,2,3-triazole-derivatives are available as commercial drugs^10^. The broad and potent activity of triazole and their derivatives has established them as pharmacologically significant scaffolds, and thus, click chemistry technique received popularity in synthesizing 1,2,3-triazole motif and it is an important tool in medicinal chemistry, which simplifies compound synthesis and also enables a modular approach to pharmacophore design for faster lead discovery and optimization^11^. The developed drug 15K significantly inhibits the oncogenic kinase PAK1 and shows inhibitory effects on different PAK1-dependent biological activities including anti-cancer, anti-inflammatory, anti-angiogenic effects, and longevity extension activities in *C. elegans*^9, 12, 13^. It shows 500 times more potent effects than ketorolac and thus received an US patent (Pub. No.: US 2017/0224830 A1, Date: Aug 10, 2017) as an effective PAK1 blocking anticancer drug. However, its druggable features, molecular properties, and binding affinity with PAK1 have not been investigated yet, and this study was undertaken with computational approaches to calculate molecular properties and simulate the binding mode of 15K with PAK1 crystals. We compared the properties of 15K with those of its precursor ketorolac and another allosteric PAK1 inhibitor (inhibitor 2) synthesized by Karpov et al.^14^. Our results reveal the strong binding properties of 15K along with its all features to be a potential anticancer drug and therefore could be a useful guideline for future research to design new and selective PAK1 inhibitors and to study PAK1 biology.

## Materials and methods

### Energy optimization of drugs

Chemical structures of ketorolac, 15K, and inhibitor 2 were drawn by Marvin Sketch (ChemAxon, Budapest, Hungary), and three-dimensional (3D) structures were generated by ChemOffice Professional 18 suite (CambridgeSoft, Cambridge, MA 02140, USA). 3D structures were then optimized by Gaussian 09 suite with Density Functional Theory (DFT), employing B3LYP/6-311G (d,p) level of theory^15^. Subsequent vibrational frequency calculations were performed after optimization to confirm that the stationary points corresponded to minima on the potential energy surface. Log files of the energy optimized drugs were uploaded in WebMO Basic (Holland, MI, USA) and GaussSum 3.0^16^ to calculate molecular electrostatic potential and density of plot (DOS). Electronic energies, enthalpies, Gibbs free energies, dipole moments, and partial charge analysis of each compound were also investigated using the same level of theory. Frontier orbital gap, hardness (*η*), softness (*S*), chemical potential (μ), electronegativity (χ), and electrophilicity index (ω) of all compounds were determined from the energies (*ε*) of frontier highest occupied molecular orbital and (HOMO) and lowest unoccupied molecular orbital (LUMO) orbitals. The ionization potential (I) and electron affinity (A) can be expressed through HOMO and LUMO orbital energies as: I = ‒ *ε*HOMO and A = ‒*ε*LUMO from Koopmans’ theorem^17^.

- Hence, Chemical potential, μ = [*ε*HOMO + *ε*LUMO]/2.
  - Hardness, *η* = [*ε*HOMO – *ε*LUMO]/2.
- The softness is defined as the reciprocal of hardness and therefore,
  - *S* = 1/*η.*
- Also, the electrophilicity index: ω = μ^2^/*2η.*

which defines a quantitative classification of global electrophilic nature of a compound^18^.

### Molecular docking analysis

Three different crystal structures of PAK1 (PDB ID: 3FXZ, 3Q52, and 5DEW) were retrieved from the RCSB protein data bank (PDB) based on the lowest resolution. PAK1 crystal structures 3FXZ, 3Q52, and 5DEW were resolved at 1.64, 1.80, and 1.90 Å, respectively. Proteins were then prepared for docking with removing water, heteroatoms, and co-crystallized ligands using Discovery Studio 4.5 (Accelrys, San Diego, CA, USA). To prepare the ligands for docking analysis, the drugs were saved as protein data bank (pdb) format after minimizing the energies with DFT using B3LYP/6-311G (d,p) level of theory. Molecular docking was employed with AutoDock Vina suite which is widely used for molecular docking as it significantly improves the accuracy of the binding mode predictions compared to its previous version AutoDock 4^19^. The grid box was prepared in the hinge region of PAK1 surrounding the active site residues, allosteric residues, gatekeeper residue, and DFG-motif residue^20, 21^. The size of the grid boxes was differed with crystal structures, and the dimension and the center of the grid boxes for each protein structure were presented in Table 1. The binding affinity of ligands was observed by kcal/mol as a unit for a negative score^19^, and the binding pose with the highest negative value was selected as the best pose for corresponding ligand. Then, the best pose was visualized and analyzed to comprehend the ligand–protein interactions through PyMOL Molecular Graphics System 2.0 (DeLano Scientific LLC, San Carlos, CA, USA) and Discovery Studio 4.5. Predicted protein–ligand interactions were validated by cross-checking with LigPlot^+^ software (obtained from https://www.ebi.ac.uk/).

**Table 1:** Grid box parameters used for docking analysis in this study.

PAK1 crystals (PDB ID)	Grid box size	
	Dimension (Å)	Center
3FXZ	X: 43.6935	X: − 17.5798
	Y: 22.5785	Y: 32.6194
	Z: 33.0583	Z: − 13.6270
3Q52	X: 38.7309	X: − 17.5988
	Y: 23.9404	Y: − 32.3660
	Z: 33.0583	Z: 10.7048
5DEW	X: 33.3401	X: 21.3679
	Y: 23.7707	Y: − 17.9706
	Z: 32.6142	Z: 13.6020

Grid box was generated in the hinge region of PAK1 crystal structures.

Three crystal structures of PAK4 (5VEF, 5XVA and 5ZJW) and PAK6 (2C30, 4KS7 and 4KS8) were also retrieved from the RCSB PDB and used them for molecular docking simulation against 15K using the similar method described above. However, in PDB, there are no full crystal structures of PAK2, PAK3, and PAK5. Therefore, homology modelling was carried out using SWISS-MODEL server (https://swissmodel.expasy.org/interactive) to predict 3D structures for PAK2, PAK3, and PAK5. Their FASTA sequences were copied from the UniProt protein data bank (https://www.uniprot.org), under the access code Q13177 (for PAK2), O75914 (for PAK3), and Q9P286 (for PAK5). PAK2, PAK3, and PAK5 consist of 524, 559, and 719 amino acid sequences, respectively. The crystal model for each protein was built based on the template showing the highest sequence identity and query coverage. Ligands were removed from the models and hydrogen atoms were added using Discovery Studio 4.5. Energy minimization was done for these three selected models with Swiss-PdBViewer prior to using for molecular docking with the similar methods described earlier.

### Molecular dynamics simulation

For validating the binding behavior of 15K with PAK1 and comparing with the same shown by inhibitor 2, molecular dynamics (MD) simulations were performed with YASARA Dynamics suite^22^ using AMBER14 force field. NPT ensemble simulation was used with normal temperature (298 K) and 1 bar of pressure. Berendsen thermostat was used to control the simulation temperature, and for short-range van der Waals and Coulomb interactions, a cut-off radius of 8.0 Å was considered. The long-range electrostatic interactions were calculated using the particle-mesh Ewald method. The periodic boundary condition was included for performing the simulation, where the cell size was 20 Å larger than the protein in all cases. These simulations were performed in water which involved the simulation of predicted model inside a trajectory box filled with solvent (including NaCl and water) and protein–ligand complex at 298 K. In order to reduce the contact area difference between the complex model and the solvent molecule, the initial model was minimized with steepest-descent algorithm. As time step, 1.25 fs was chosen, and 100 ns MD simulation was performed for each system where trajectories were saved after every 100 ps. The MD trajectories were analyzed by a macro program written in YANACONDA language. In this analysis, the root mean square deviation (RMSD) was computed for the protein backbone and residues with a view to checking the stability of the trajectories. In order to study the flexibility of the trajectories, the root mean square fluctuation (RMSF) was calculated per residue. Further analysis was performed by calculating the radius of gyration (Rgyration) and solvent accessible surface area (SASA) of the enzyme within the 100 ns of production time.

### Principal component analysis

The principal component analysis (PCA) was applied for analyzing the structural and energy fluctuations among apo PAK1 and ligand-PAK1 complexes. It considers different multivariate energy factors to project into low-dimensional planetary^23, 24^, which manifests the variability exist in MD trajectories. The bond distances, bond angles, dihedral angles, planarity, van der Waals energies, and electrostatic energies represented structural and energy profile. The PCA analysis assisted to reflect the variations among different groups. The 100 ns trajectory data were pre-processed using centering and scaling for the analysis. The multivariate factors were arranged in the X matrix and expressed them into a product of two new matrices by the following equation:

$$X={T}_{k}{P}_{k}^{T}+E$$

where Tk, matrix of scores symbolizes how sample is relative to each other; Pk, matrix of loadings manifests how variables correlate to each other, k is the number of factors integrated in the model and E is the matrix of residuals. The analysis was performed using R programming language and the plots were visualized by ggplot2 package^25^.

### Multiple receptor conformers-based molecular docking

Two methods were used to generate multiple conformers of PAK1 crystal. Firstly, the crystal structure of PAK1 (PDB ID: 3Q52) was used in 100 ns MD by YASARA Dynamics program as stated above. Then, 100 snapshot conformers were generated and saved in PDB format after every 5 ns of 100 ns MD simulation. In another approach, 20 crystallographic structures of PAK1, except for 3Q52, were collected from the RCSB PDB database (PDB ID: 1YHV, 1YHW, 3FY0, 3Q53, 4DAW, 4EQC, 5DFP, 3Q4Z, 4O0R, 4O0T, 4P90, 4ZJI, 4ZJJ, 4ZLO, 4ZY4, 4ZY5, 5DEY, 6B16, 5KBR, 5IME). Flexible docking with 15K against the multiple conformers of the PAK1 was performed and subsequently analyzed using the same protocol by AutoDock Vina. Inhibitor 2 was also docked against multiple conformers in this experiment to compare its binding affinity with that of the 15K.

### Prediction of ADMET properties

Pharmacokinetic parameters related to drug absorption, distribution, metabolism, excretion, and toxicity of 15K were predicted with pKCSM online tool^26^ and compared to the predicted values of inhibitor 2. pKCSM is a novel approach to predict pharmacokinetic and toxicology parameters using graph-based signatures, which develop and implement 14 quantitative regression models with actual numeric outputs and 16 predictive classification models with categorical outputs for predicting a wide range of ADMET properties for novel diverse molecules. We also used ADMETlab 2.0 (https://admetmesh.scbdd.com/) to compare the ADMET results.

### Prediction of bioactivities, physicochemical properties and druglikeness of 15K

Prediction of activity spectra for substances (PASS) program was utilized to predict biological activities of 15K and inhibitor 2. It is an online-based computer program for the evaluation of overall biological potentials according to the structure–activity relationship (SAR)^27^. Through this program, prediction is carried out by comparing the desired molecules with a training set including more than 205,000 compounds revealing more than 7200 types of biological activities^28^. This tool predicts a compound with its probability to be active (Pa) and a probability to be inactive (Pi). The easy, efficient, and login-free web tool SwissADME^29^ was here used to predict the physicochemical properties and druglikeness of 15K.

### In vitro toxicity evaluation of 15K

Cytotoxic effects of 15K were evaluated with two different approaches. Firstly, using the normal fibroblast 3T3L1 cells, cytotoxicity was investigated with MTT assay^30^. The cells were seeded for overnight in a 24-well plate with the density of 2.5 × 10^4^ cells/well and then treated with 15K at different concentrations ranging up to 20 µM for 48 h. After that, the cells were washed with ice-cold PBS, and an aliquot of MTT solution (0.5 mg/ml in PBS) was added to each well. After incubating the cells at 37 °C in humidified conditions, formazan crystals formed in each well were dissolved with 400 μL dimethyl sulfoxide (DMSO), and the plate was shaken for 10 min at room conditions. The absorbance was measured at 570 nm wavelength using a microplate reader. Cytotoxicity was calculated based on the absorbance of the treated versus untreated control cells. In the second approach, toxic effects of 15K were evaluated using a small animal model brine shrimp (*Artemia salina*)^31^. Brine shrimp eggs (200 mg, Tetra Brine Shrimp Eggs, Spectrum Brands Holdings, Inc., Kanagawa, Japan) was hatched in artificial seawater supplemented with dried yeast with suitable aeration in room conditions for 48 h. Active brine shrimp nauplii were then transferred to a glass petri dish-containing artificial seawater and then transferred to each well of a 96-well plate. Each well was contained 10–15 larvae in 100 μl artificial seawater. 15K dissolved in DMSO was added in each well except for the control wells in different concentrations, and then the well plate was incubated at 20 °C. The final concentration of DMSO in the treated wells was 1% (v/v), and DMSO at 1% was used in the control wells. The number of dead larvae in each well was counted using magnifying lenses (5 × or 10 ×) after 24 h and 48 h of incubation. Finally, methanol (50 μl) was added to each well, and the total number of larvae in each well was counted after 1 h. The survival percentage of brine shrimps for each concentration was then calculated.

### Protein–protein interaction (PPI) network and enrichment analysis

PPI network was prepared using the default options of STRING database and then it was imported to the Cytoscape for further analysis. Total number of nodes and edges were 11 and 50, respectively. Enrichment analysis was also done with the STRING database, which shows a total of 235 gene ontology (GO) terms (component/process/function) enrichment and 38 KEGG pathway enrichment. Bubble plots were prepared using 20 highly enriched GO terms and KEGG pathways.

## Results

### Geometry optimization and the features of frontier molecular orbitals

**Figure 1:**
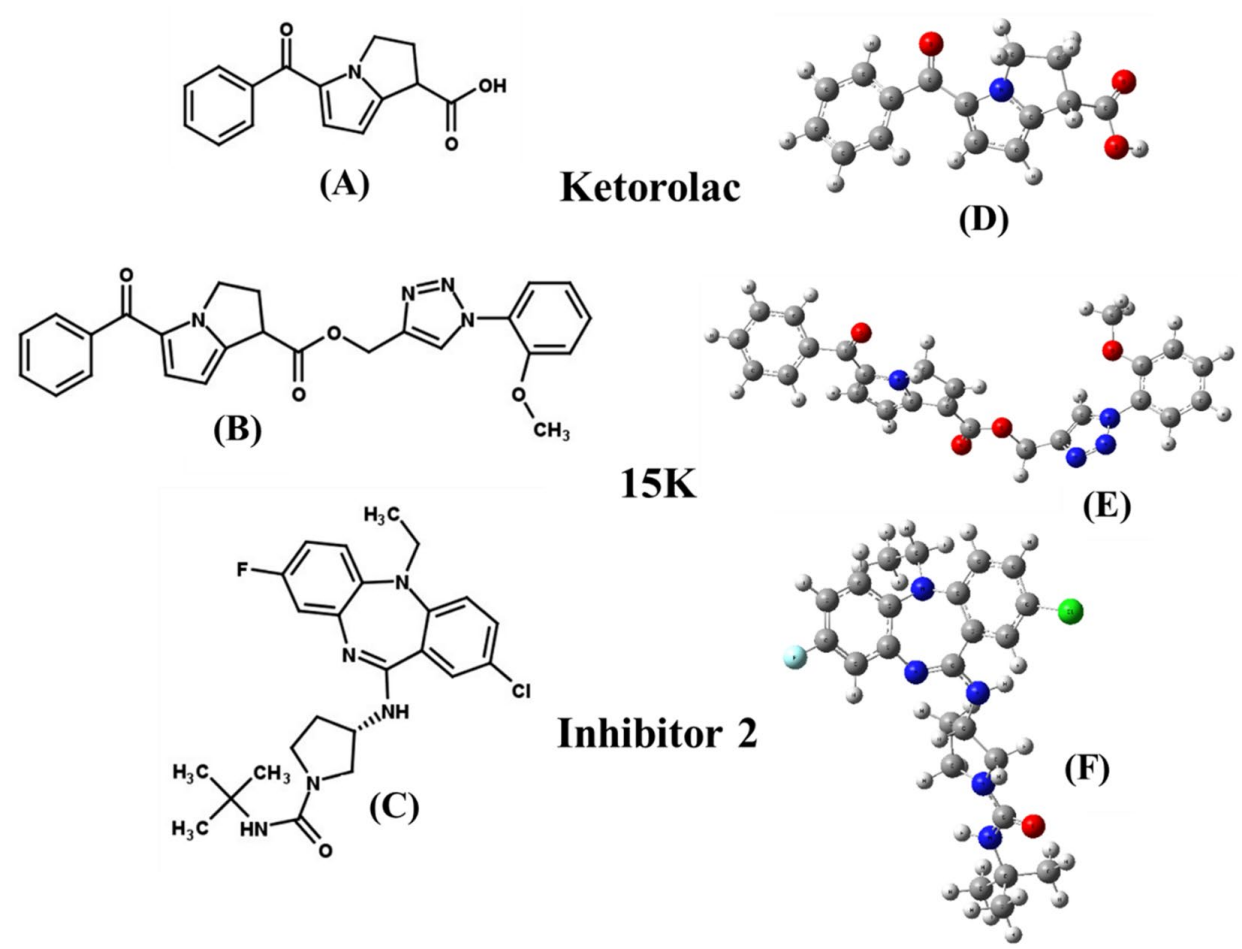
Chemical structures (**A**–**C**) and the most stable optimized forms (**D**–**F**) of ketorolac, 15K, and inhibitor 2.

Full geometry optimization of ketorolac, 15K, and inhibitor 2 was carried out by DFT calculation, and their most stable optimized structures are presented in Fig. 1. Table 2 represents the electronic energy, enthalpy, Gibbs free energy, and dipole moment of the compounds. 15K shows different structural and thermodynamic properties compare with its precursor ketorolac. It exhibits higher electronic energy, enthalpy, and Gibbs free energy than ketorolac. Dipole moment is also higher in 15K than ketorolac and inhibitor 2, demonstrating that the polarity of 15K is higher than both other drugs.

**Table 2:** Electronic energy, enthalpy, Gibbs free energy (in Hartree), and dipole moment (Debye) of 15K, ketorolac, and inhibitor 2.

Name	Electronic energy	Enthalpy	Gibbs free energy	Dipole moment (Debye)
15K	**− **1484.47	**− **1484.47	**− **1484.57	6.17
I-2	**− **1840.91	**− **1840.91	**− **1841.00	3.30
Ket	**− **859.81	**− **859.81	**− **859.87	3.96

*Ket* Ketorolac, *I-2* Inhibitor 2.

**Figure 2:**
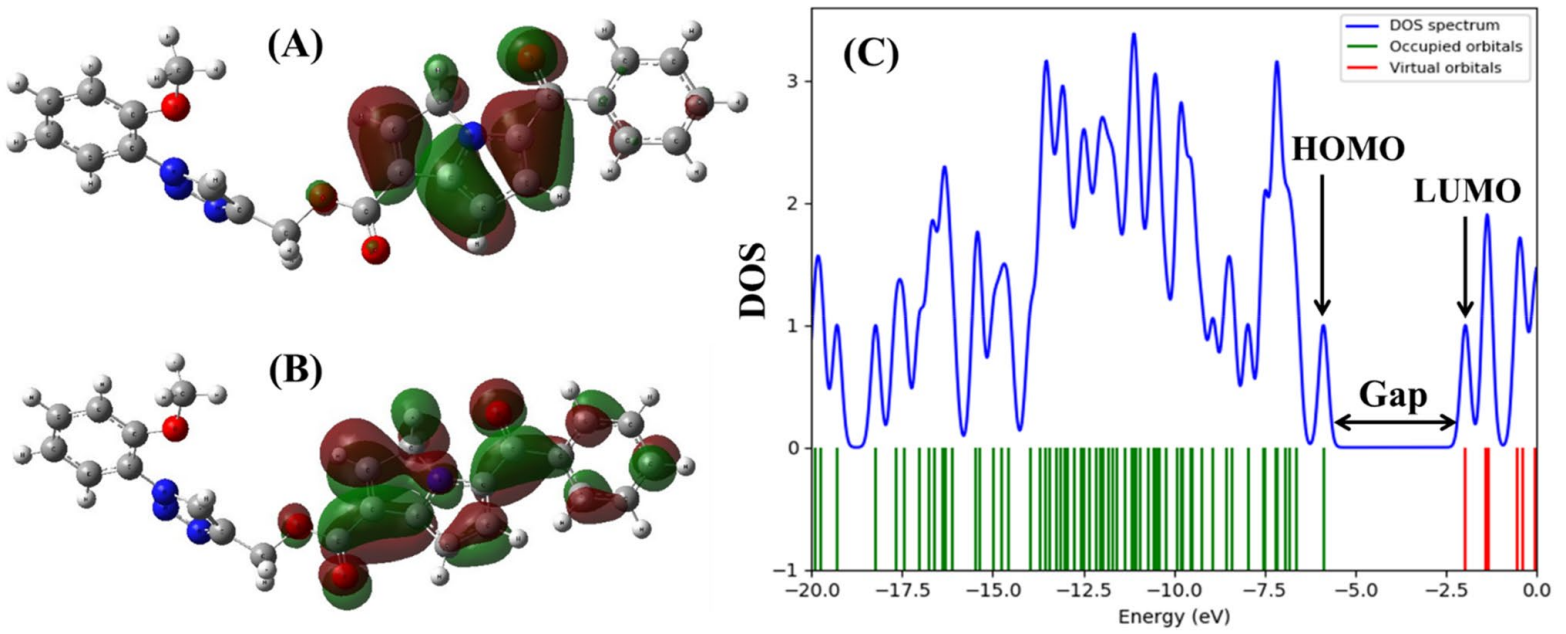
Molecular orbital distribution plots of HOMO and LUMO in the ground state of 15K at DFT/B3LYP level of theory in the gas phase (**A**, **B**). Density of state (DOS) plot (**C**) showing the HOMO–LUMO energy gap of 15K.

Frontier molecular orbital analysis reveals that 15K has the lower HOMO–LUMO energy gap (0.144 Hartree) compared with ketorolac and inhibitor 2, and in turn 15K shows low hardness and high softness value (Table 3). Figure 2 represents the molecular orbital distribution in 15K and the energy gap between HOMO and LUMO. The electron distribution in both HOMO and LUMO is observed in the precursor molecule not in its triazole moiety.

**Table 3:** Results of quantum chemical calculations of 15K, ketorolac, and inhibitor 2.

Name	^ε^HOMO	^ε^LUMO	Gap	μ (chemical potential)	*η *(hardness)	*S *(softness)	Electronegativity (x)	Electrophilicity Index (ω)
15K	**− **0.216	**− **0.072	0.144	0.144	0.072	13.90	0.144	0.144
I-2	**− **0.208	**− **0.058	0.151	0.133	0.075	13.30	0.133	0.118
Ket	**− **0.227	**− **0.062	0.166	0.145	0.083	12.06	0.145	0.127

*Ket* Ketorolac, *I-2* Inhibitor 2; All values are expressed in Hartree.

**Figure 3:**
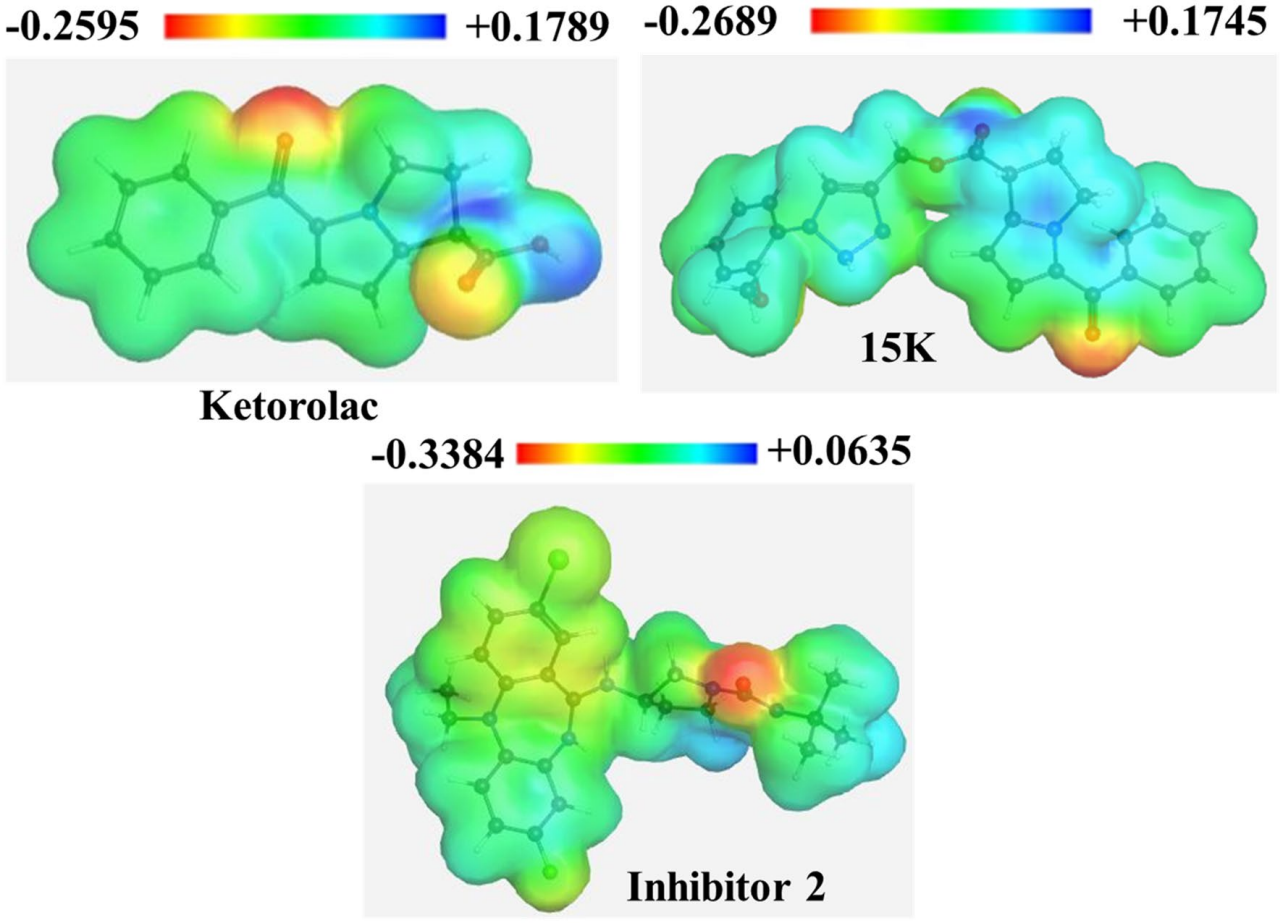
Molecular electrostatic potential map of ketorolac, 15K, and inhibitor 2.

Molecular electrostatic potential is highly informative concerning the nuclear and electronic charge distribution of the molecules. It is a tool for interpretation and prediction of chemical reactivity. We calculated molecular electrostatic potential (MEP) to predict the reactive sites for electrophilic and nucleophilic attack of all three optimized structures. Red color indicates maximum negative area (favorable site for electrophilic attack), blue color represents the maximum positive area (favorable site for nucleophilic attack) and zero potential area is represented by green color (Fig. 3). Here, 15K potentially shows negative − 0.2689 a.u. and positive + 0.1745 a.u.

### Molecular interactions of drugs with different PAK isoforms

Molecular docking analysis shows that 15K can strongly bind with all the three PAK1 crystals (3FXZ, 3Q52, and 5DEW) compared with ketorolac and inhibitor 2. The binding affinities for ketorolac were ranging − 6.7 to − 7.0 kcal/mol, while the binding affinities for 15K were − 8.6 to − 9.2 kcal/mol (Table 4). 15K exhibited higher binding energies than those shown by inhibitor 2.

**Table 4:** Binding affinities and molecular interactions of 15K, ketorolac, and inhibitor 2 with three different crystal structures of PAK1.

Drug	PAK1 crystals	Binding affinity (Kcal/mol)	Hydrogen bond interaction		Hydrophobic interaction		Electrostatic interaction	
			Residues	Distance (Å)	Residues	Distance (Å)	Residues	Distance (Å)
15K	3FXZ	**− **8.6	**Glu315**	2.5	Ala297	4.0	–	–
			**Glu315**	2.4	Leu396	4.6	–	–
			Asp407	2.5	Lys308	5.3	–	–
			–	–	Leu311	4.8	–	–
			–	–	Val284	4.6	–	–
	3Q52	**− **9.2	**Glu315**	3.0	Phe410	5.6	–	–
			**Glu315**	1.9	Ala297	3.9	–	–
			Ser281	2.3	Leu396	4.8	–	–
			–	–	Leu311	5.0	–	–
	5DEW	**− **8.9	**Glu315**	2.3	Ile276	3.5	**Glu315**	3.6
			Lys299	3.0	Val342	3.8	–	–
			**Glu315**	2.4	Leu396	3.9	–	–
			–	–	Tyr346	5.0	–	–
			–	–	Ile316	5.4	–	–
			–	–	Met344	5.3	–	–
			–	–	Ala297	4.6	–	–
Ket	3FXZ	− 6.9	–	–	Arg299	3.6	Arg299	4.4
			–	–	Arg299	5.2	–	–
			–	–	Met344	4.8	–	–
			–	–	Ala297	3.5	–	–
			–	–	Leu396	5.2	–	–
	3Q52	− 6.7	Thr406	2.4	Leu396	3.7	–	–
			–	–	Leu396	3.9	–	–
			–	–	Val284	4.7	–	–
			–	–	Ala297	4.1	–	–
	5DEW	− 7.0	Lys299	2.0	Val342	3.6	**Glu315**	3.7
			–	–	Met344	3.5	–	–
			–	–	Ile316	5.4	–	–
			–	–	Met344	5.2	–	–
I-2	3FXZ	**− **7.9	Arg299	3.5	Ile276	4.3	–	–
			–	–	Val284	3.5	–	–
			–	–	Leu396	4.3	–	–
			–	–	Val284	4.4	–	–
	3Q52	**− **7.8	Gln278	2.5	Val284	3.7	Asp407	5.3
			Arg299	2.6	Val284	3.9	–	–
			Arg299	2.3	Val284	4.8	–	–
			Asp407	2.8	Ala297	3.9	–	–
			–	–	Arg299	4.6	–	–
			–	–	Met344	4.4	–	–
			–	–	Ala297	5.0	–	–
			–	–	Val284	4.5	–	–
	5DEW	**− **7.4	**Glu315**	2.4	Met319	4.9	LYS299	3.6
			–	–	Met344	4.5	LYS299	4.4
			–	–	Val284	5.3	–	–
			–	–	Ala297	5.0	–	–
			–	–	Lys299	4.6	–	–
			–	–	Met344	4.6	–	–

*Ket* ketorolac, *I-2* Inhibitor 2.

**Figure 4:**
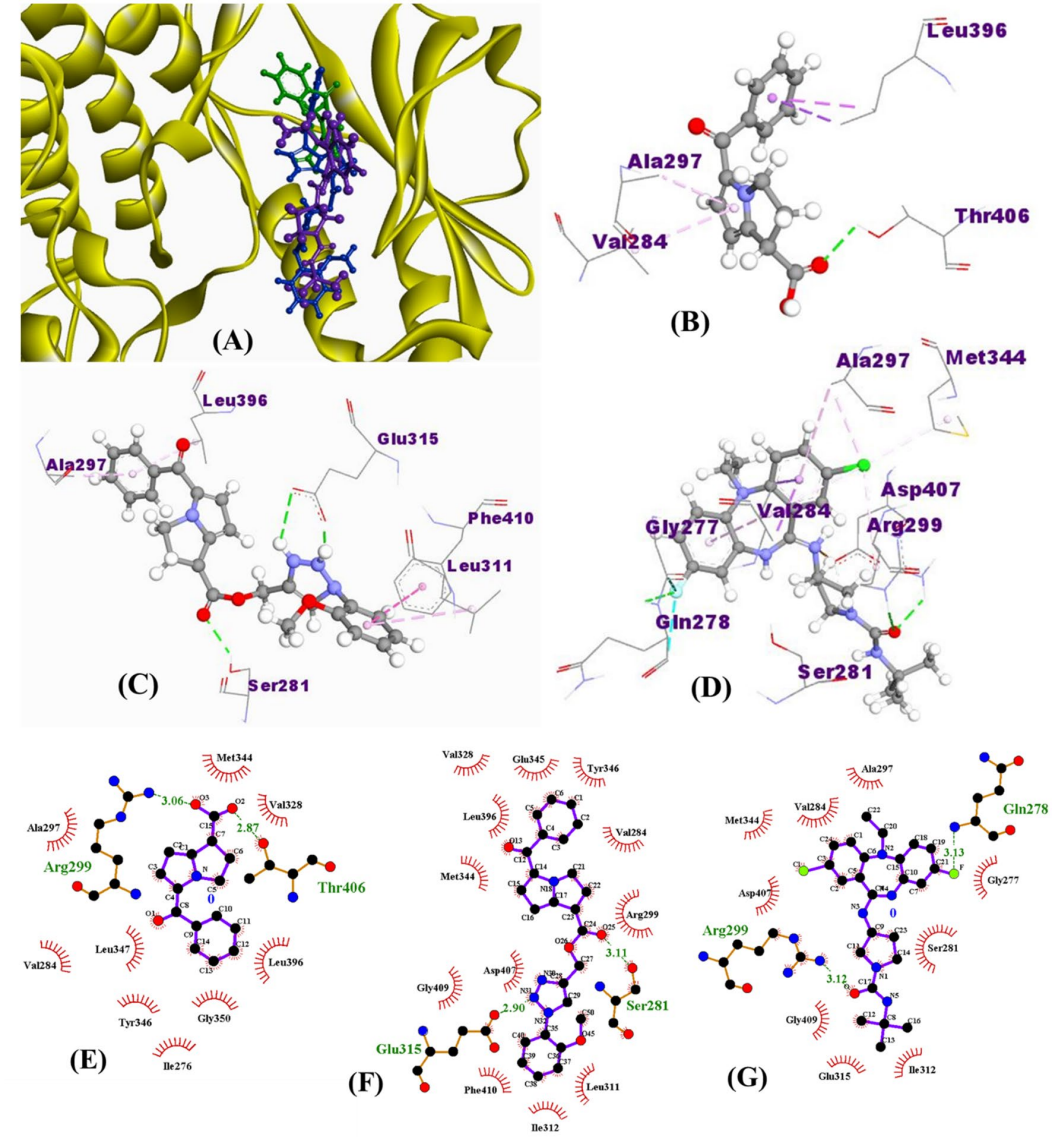
Binding orientation of ketorolac (green), 15K (blue), and inhibitor 2 (magenta) in the catalytic sites of PAK1 (PDB ID: 3Q52, Resolution: 1.80 Å) (**A**). Non-bonding interactions of ketorolac (**B**), 15K (**C**), and inhibitor 2 (**D**) with different PAK1 residues as determined by Discovery Studio 4.5. Green dashed lines in **B**, **C**, and **D** represent hydrogen bond interaction. Molecular interactions of ketorolac (**E**), 15K (**F**), and inhibitor 2 (**G**) with PAK1 residues were cross-checked by LigPlot+. PAK1 residues with green color text in **E-G** are responsible for hydrogen bond formation with the ligand.

**Figure 5:**
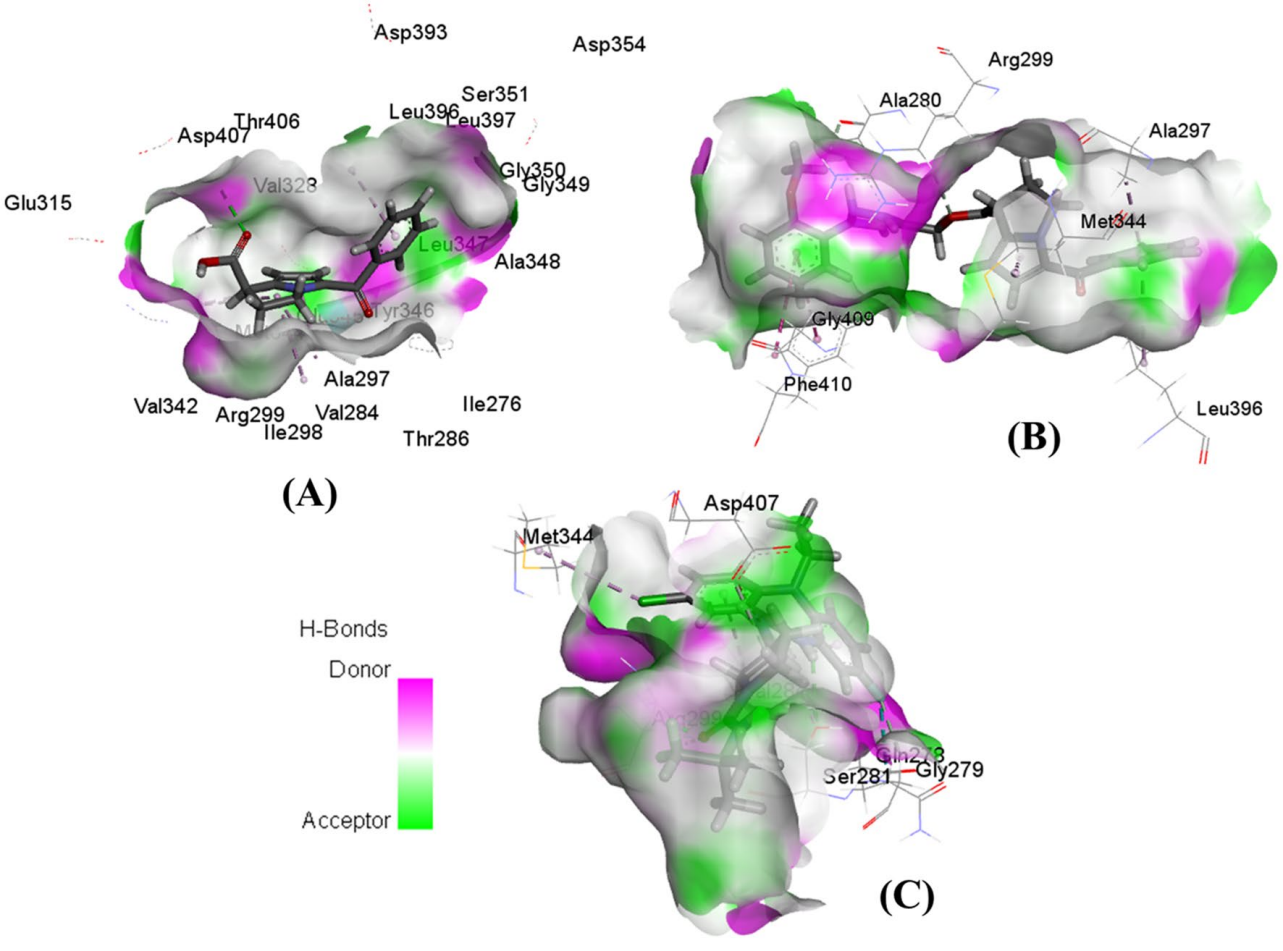
Hydrogen bond surface representations of PAK1 in complex with three ligands. Interaction of ketorolac (**A**), 15K (**B**), and inhibitor 2 (**C**) with PAK1.

15K interacted with all three PAK1 crystals by forming three hydrogen bond interactions, while ketorolac formed only one hydrogen bond with both 3Q52 and 5DEW but no hydrogen bond with 3FXZ (Fig. 4). However, inhibitor 2 interacted differentially with different crystals forming one hydrogen bond with 3FXZ and 5DEW and four hydrogen bonds with 3Q52. In every case, 15K formed two hydrogen bonds with the αC helix residue Glu315 having the bond distance 2.5 Å or less. It also showed another hydrogen bond interaction with the DFG residue Asp407 of 3FXZ. One electrostatic interaction with Glu315 was observed in 15K-5DEW complex. The hydrogen bond interactions observed by ketorolac were not with any active site or even allosteric residues, while it showed an electrostatic interaction with Glu315 of 5DEW. Inhibitor 2 showed a hydrogen bond interaction with Glu315 of 5DEW crystal and DFG residue Asp407 of 3Q52. Both ketorolac and inhibitor 2 formed hydrophobic interaction with the gatekeeper residue Met344 of two crystals, however 15K only showed this interaction with one crystal. Moreover, hydrogen bond surface shows that the residues such as Ser281, Arg299, Asp407, Gly279, and Ala280 can create strong donor regions on the drug-protein interaction surface (Fig. 5).

When 15K was docked against other PAK isoforms, PAK2–6, it did not show stronger binding affinities than what it showed for PAK1. It only showed better binding energies with the modelled PAK2 crystal (**− **8.4 kcal/mol) and one PAK4 crystal (**− **8.3 kcal/mol) (Tables 5 and 6). The binding affinities with other PAK isoform crystals were low (**− **0.8 to − 7.7 kcal/mol). Most importantly, 15K did not show any hydrogen bond interaction with the active site residues or allosteric residues of PAK2–6, even no hydrophobic and electrostatic interactions were observed with those residues. Ligand–protein molecular interaction results from LigPlot^+^ coincide with those obtained from Discovery Studio (Fig. 4E–G).

**Table 5:** Binding affinities and molecular interactions of 15K with three different crystal structures of PAK4 and PAK6.

PAKs	PDB ID	Binding affinity (Kcal/mol)	Hydrogen bond interaction		Hydrophobic interaction		Electrostatic interaction	
			Residues	Distance (Å)	Residues	Distance (Å)	Residues	Distance (Å)
PAK4	5VEF	**− **8.3	Asp458	2.4	Ile327	3.9	–	–
			Glu329	2.9	Val335	3.9	–	–
			–	–	Ala402	3.8	–	–
			–	–	Leu447	3.5	–	–
			–	–	Val335	4.5	–	–
			–	–	Leu447	5.3	–	–
			–	–	Ala348	4.5	–	–
	5XVA	**− **7.7	Asp458	2.8	Leu447	3.5	–	–
			–	–	Phe397	4.9	–	–
			–	–	Ile327	5.1	–	–
			–	–	Ile327	5.3	–	–
			–	–	Ile337	5.4	–	–
			–	–	Val335	4.1	–	–
			–	–	Leu447	5.3	–	–
			–	–	Val335	5.1	–	–
			–	–	Ala348	4.0	–	–
	5ZJW	− 5.9	Lys345	2.2	Lys345	5.1	GLU396	4.3
			Leu346	2.1	–	–	GLU399	3.9
			Arg453	2.1	–	–	–	–
			Glu399	2.2	–	–	–	–
PAK6	2C30	− 5.0	Ile413	2.4	Ile413	3.6	–	–
			Lys436	2.9	Leu533	3.9	–	–
			–	–	Leu484	5.4	–	–
			–	–	Val421	4.9	–	–
	4KS7	− 5.8	Phe483	2.9	Thr535	3.5		
			Phe483	2.4	Arg431	4.4		
			Arg431	2.1	–	–	–	–
			Gln432	2.9	–	–	–	–
			Asp537	2.2	–	–	–	–
			Arg431	3.6	–	–	–	–
	4KS8	− 0.8	Arg539	2.0	–	–	Arg431	3.7
			Arg431	2.6	–	–	Arg539	4.2
			Leu484	3.1	–	–	–	–

**Table 6:** Binding affinities and molecular interactions of 15K with the modelled structures of PAK2, PAK3 and PAK5.

PAK model	Binding affinity (Kcal/mol)	Hydrogen bond interaction		Hydrophobic interaction		Electrostatic interaction	
		Residues	Distance (Å)	Residues	Distance (Å)	Residues	Distance (Å)
PAK2	− 8.4	Asp372	2.90	Val263	3.78	Asp372	3.76
		Asp372	2.63	Thr332	3.96	–	–
		Gln257	2.31	Thr332	3.88	–	–
		Asp372	2.45	Leu375	3.69	–	–
		Gln257	2.43	Ile255	5.07	–	–
		Gly256	3.69	Leu375	4.76	–	–
		–	–	Leu444	5.43	–	–
		–	–	Ala276	5.27	–	–
PAK3	− 4.6	Glu323	2.52	Ile326	3.80	–	–
		Gln426	2.82	Leu330	4.92	–	–
		Glu323	3.06	Ile326	4.26	–	–
		–	–	Lys269	4.53	–	–
		–	–	–	–	–	–
		–	–	–	–	–	–
		–	–	–	–	–	–
		–	–	–	–	–	–
		–	–	–	–	–	–
PAK5	− 7.5	Leu526	2.42	Ile455	3.94	–	–
		Phe525	3.51	Leu575	3.58	–	–
		–	–	Val463	5.00	–	–
		–	–	Lys478	4.81	–	–
		–	–	Met523	4.44	–	–
		–	–	Ala476	4.66	–	–
		–	–	Ala476	5.05	–	–
		–	–	Val507	5.02	–	–
		–	–	Met523	4.67	–	–
		–	–	Val463	5.37	–	–
		–	–	Leu575	5.30	–	–
		–	–	Ala530	5.29	–	–

### Analysis of molecular dynamics simulation

**Figure 6:**
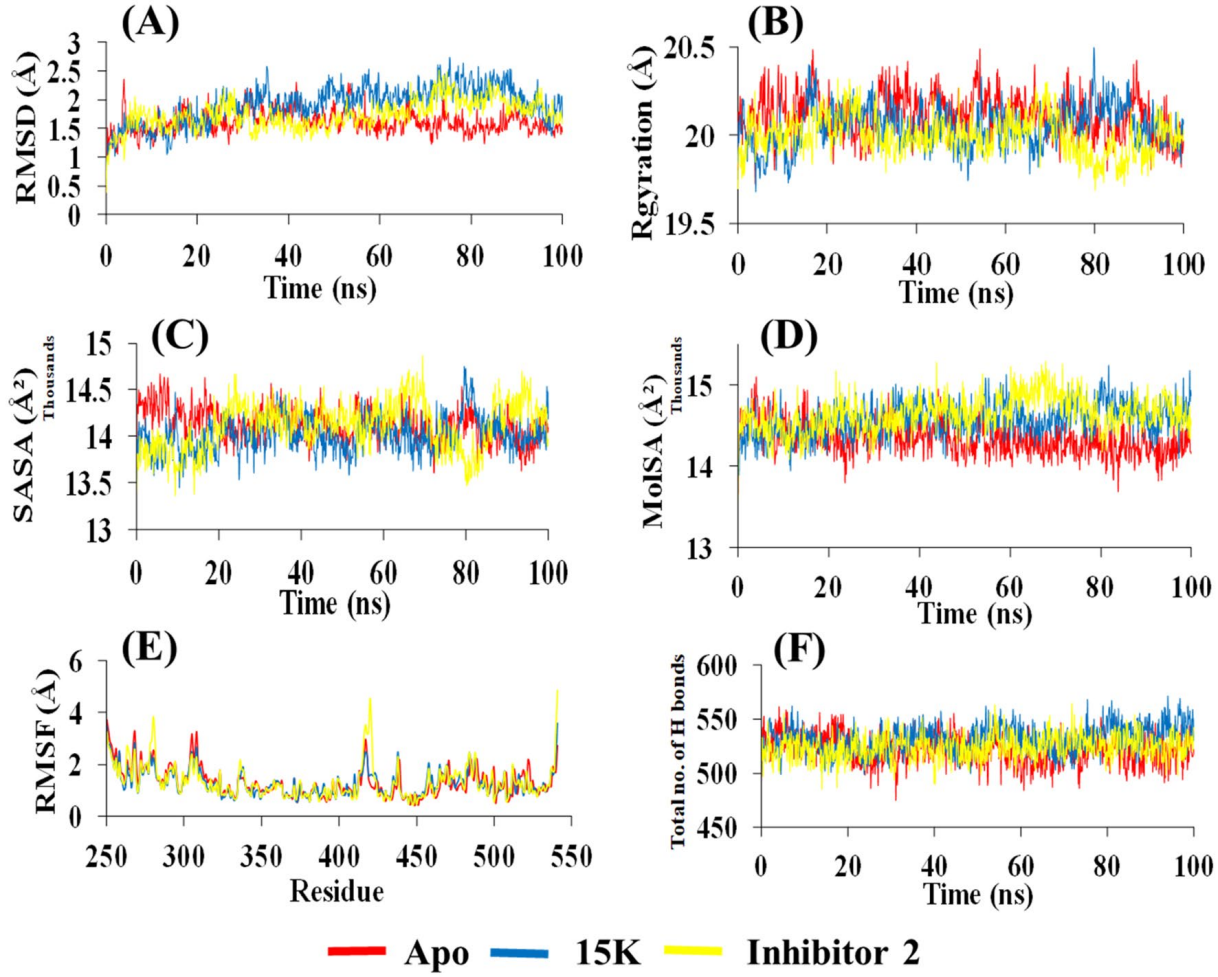
Results of 100 ns MD simulation of PAK1 in complex with 15K and inhibitor 2. (**A**) Root mean square deviation (RMSD) values of C-α atom of PAK1 in the complexes with 15K and inhibitor 2. The structural changes of PAK1 by means of radius of gyration, solvent accessible surface area, molecular surface area, root means square fluctuations (RMSF), and total number of hydrogen bonds formed during the simulation are presented in (**B**, **C**, **D**, **E**, and **F**), respectively.

We carried out MD simulation and explored the stability of each MD system to determine the promising PAK1 inhibitors. MD simulation for each complex of PAK1 with two ligands (15K and inhibitor 2) is performed for 100 ns. We also conducted an MD run for PAK1 apo which shows stability for RMSD value during the whole run (average 1.6 Å). Although the RMSDs (0.4–2.7 Å) for α-carbon atoms is slightly higher in 15K than inhibitor 2 (0.3–2.5 Å), our results still suggest that 15K can be stable in physiological conditions. RMSD values of 15K are slightly increased to 2.7 Å after 75 ns and show few smaller fluctuations at 35 and 73 ns (Fig. 6A). The trajectories generated otherwise during the whole run are found to be stable. Thus, higher structural stability in the PAK1-15K complex is detected. Inhibitor 2 exhibits high fluctuations from 73 to 74 ns, but is stable in the later phase with the average of 1.7 Å. The average RMSD for 15K is 1.9 Å, indicating that it can be a stable and potent inhibitor.

The Rgyration is a parameter that indicates structural compactness of protein. A lower degree of fluctuation with its consistency throughout the simulation run suggests the greater compactness and rigidity of the system^32^. A similar Rgyration is detected for apo (average 20.11 Å), 15K (average 20.05 Å), and inhibitor 2 (average 19.99 Å), which suggests comparatively tight packing of the ligand–protein complex during the whole run. This result indicates that the complexes are relatively compact and not much changed after ligand binding (Fig. 6B). The expansion of protein volume is indicated by higher SASA value and a low fluctuation is expected over the simulation time. SASA shows the lowest value for 15K-PAK1 complex (average 14,013 Å^2^) compared to inhibitor 2 (average 14,106 Å^2^) (Fig. 6C). Molecular surface area (MolSA) was evaluated for both ligand–protein complexes. Inhibitor 2 complex shows the highest MolSA (average 14,649 Å^2^). 15K and apo show slightly lower value of MolSA with the average of 14,571 Å^2^ and 14,339 Å^2^, respectively (Fig. 6D).

RMSF property is highly used to capture the conservation of protein dynamics^33^. During the protein–ligand interaction, a significant fluctuation of RMSF values of residues (415–420) is observed (Fig. 6E). In 15K-PAK1 complex, most of the residues show a low RMSF (average 1.29 Å) than inhibitor 2-PAK1 complex (average 1.34 Å) or the apo form (average 1.31 Å). The RMSF of hotspot residues Glu315, Met344, and Asp407 in 15K-PAK1 complex is lower than inhibitor 2-PAK1 complex, reflecting that 15K can stimulate more strong interactions with these key residues^34^. Furthermore, the visual analysis of MD simulation trajectories suggests that both ligands engaged in significant binding interactions with the hotspot residues mentioned earlier.

**Figure 7:**
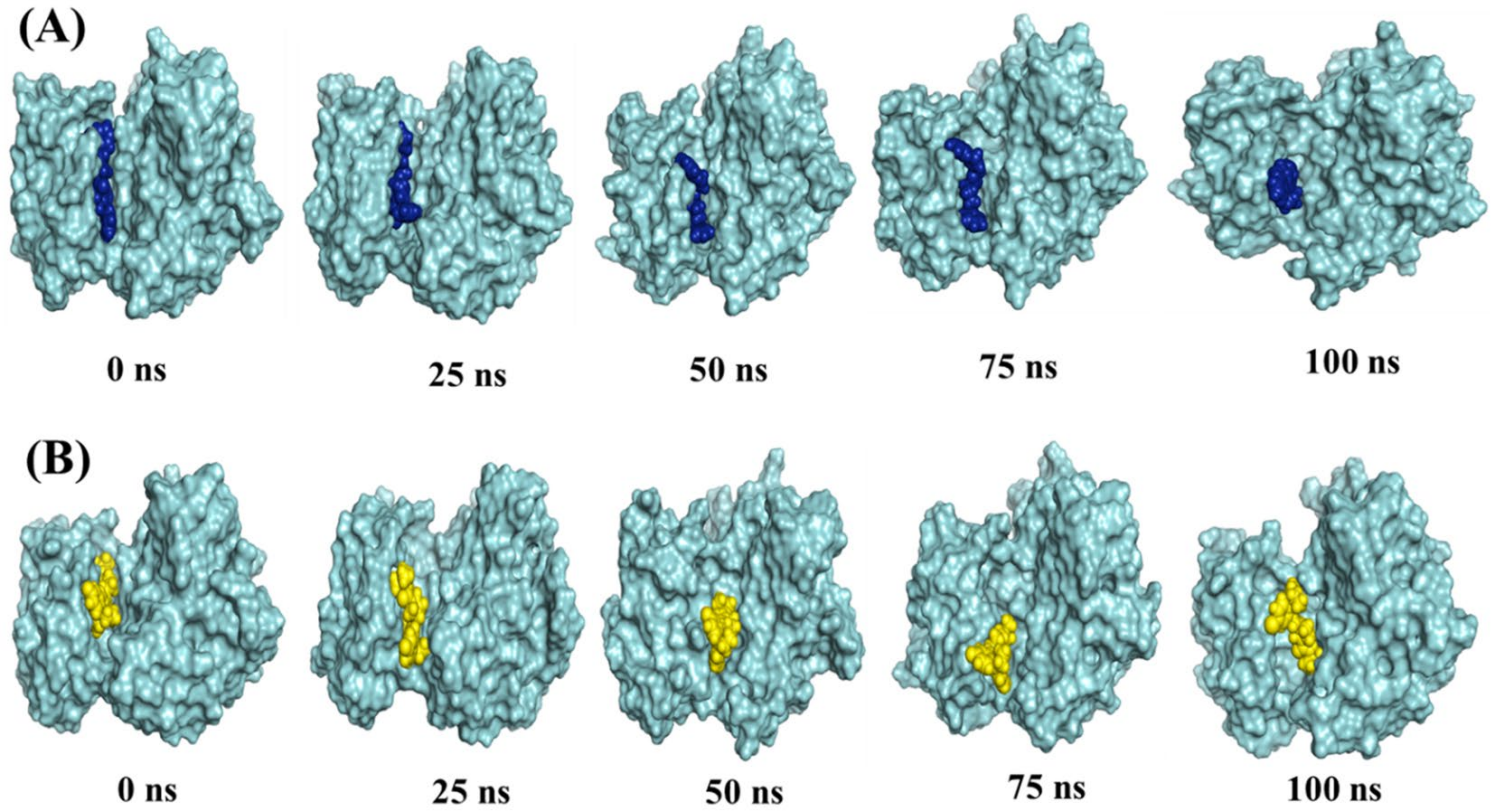
Binding poses of 15K and inhibitor 2 over the course of 100 ns MD simulation. The crystal structure of PAK1 is shown as cyan surface with 15K (Blue) and inhibitor 2 (Yellow) as spheres in (**A**) and (**B**), respectively.

Stability of the protein molecule largely depends on hydrogen bonds. The intermolecular hydrogen bond formed during the whole 100 ns run is observed and the maximum number of hydrogen bond is predicted for 15K (average 531), whereas the number of hydrogen bond in inhibitor 2-PAK1 and apo is almost similar (523 and 521) (Fig. 6F). The snapshot of conformers displays that both ligands were mostly in the hinge region of PAK1 throughout the entire simulation process (Fig. 7A,B).

### Principal component analysis on MD simulation

**Figure 8:**
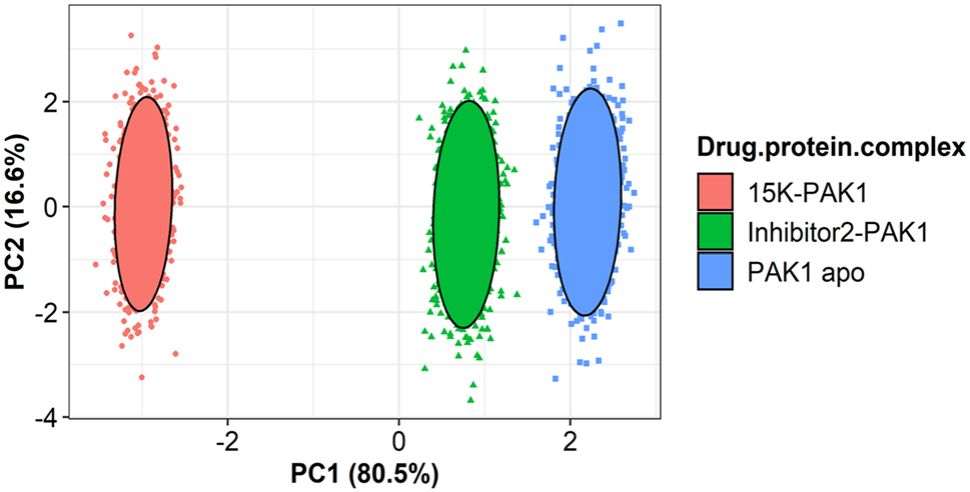
PCA analysis on MD simulation. The score plot displays three clusters, where each dot indicates one time point. The clustering is attributable to: 15K-PAK1 (Red), inhibitor 2-PAK1 (Green), and apo-PAK1 (Blue).

The PCA model was built to evaluate the structural and energy data retrieved from MD simulation analysis. There are three training sets accounted for the analysis. For the PCA model, total 97.1% of the variance is expressed by PC1 and PC2, wherever PC1 exposes 80.5% and PC2 exposes 16.6% of the variance (Fig. 8). The score plot shows different clusters for apo-PAK1 (Blue), 15K-PAK1 complex (Red) and inhibitor 2-PAK1 complex (Green). The fluctuations during MD simulation are responsible for different cluster formation among three groups. Here, 15K-PAK1 complex exhibits significant differences than both apo-PAK1 and inhibitor 2-PAK1 complex. The divergent behavior of 15K-PAK1 complex infers that the complex has exerted *s*ubstantial variations in the structural and energy profile, which may favor the strong interaction of 15K with PAK1.

### Multiple receptor conformers based molecular docking

**Figure 9:**
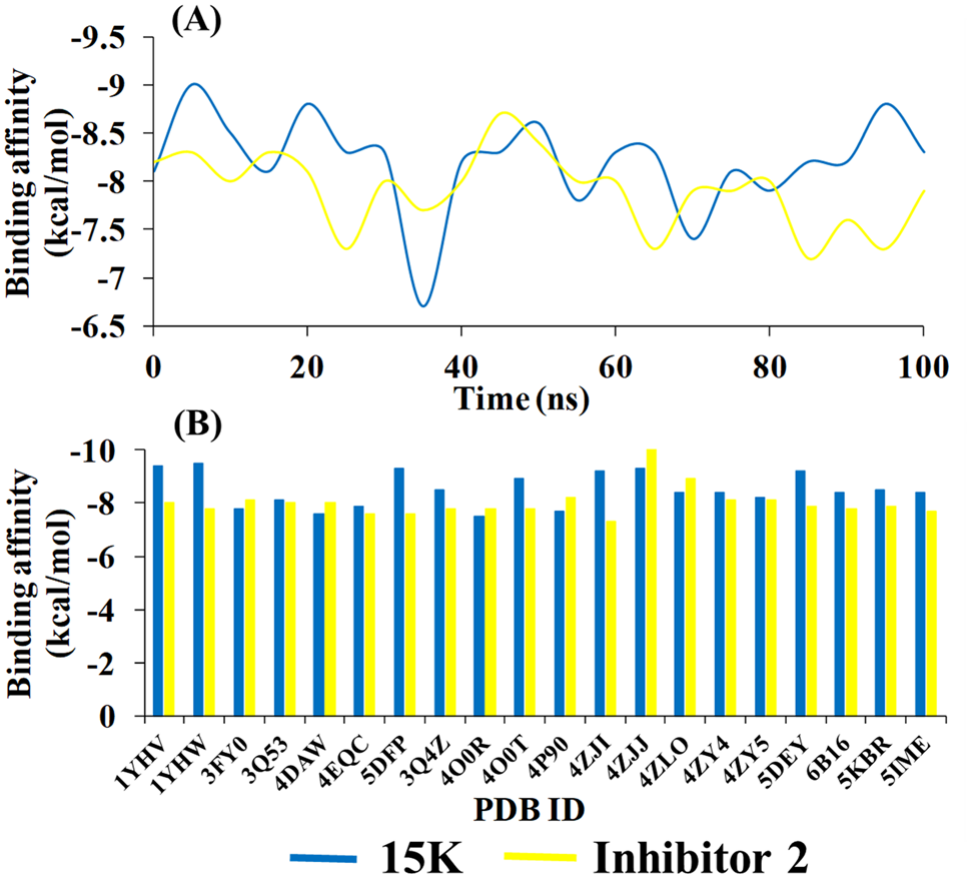
Binding energy values of 15K and inhibitor 2 docked against multiple PAK1 snapshot conformers generated by 100 ns MD simulation (**A**) and PAK1 conformers retrieved from the RCSB protein data bank (**B**).

Two types of PAK1 conformers generated from MD simulation and PDB were used to detect the difference in binding affinity of 15K and inhibitor 2 (Fig. 9). Figure 9A represents that 15K exhibits more binding affinity than inhibitor 2 against 20 different MD trajectories (0.1, 5, 10, 15,… 0.100 ns) obtained from 100 ns run. Inhibitor 2 demonstrates an average binding affinity of − 7.9 kcal/mol, whereas 15K shows − 8.2 kcal/mol. Similarly, the binding affinity was higher for 15K than that for inhibitor 2 when they were docked with 20 crystal structures of PAK1 other than 3Q52 (Fig. 9B).

### ADMET, physicochemical, and pharmacological properties, and druglikeness of 15K

To screen the most effective compound during drug development process, different factors like absorption, distribution, metabolism, excretion, and toxicity (ADMET) play important roles. Through an in silico ADMET analysis, 15K shows poor aqueous solubility (log mol/L =  − 4.26) (Table 7), indicating poor absorption via oral administration. The ADMET table (Table 7) also expresses that 15K could be distributed in blood stream only and is not able to cross the blood brain barrier (BBB) and in turn it could not pose any psychological side effect.

**Table 7:** Predicted ADMET (Absorption, distribution, metabolism, excretion, and toxicity) properties of 15K.

Properties	Model name	Predicted value	
		pKCSM results	ADMETlab 2.0 results
Absorption	Water solubility (log mol/L)	− 4.26	− 5.28
	Human intestinal absorption (% Absorbed)	99.44	High
	Caco2 permeability (log Papp in 10-6 cm/s)	1.10	− 4.967
Distribution	Volume of distribution (human) (log L/kg)	0.06	0.542
	BBB permeability (logBB)	− 1.12	− 1.12
Metabolism	CYP2D6 substrate	No	No
	CYP3A4 substrate	Yes	Yes
	CYP1A2 inhibitor	Yes	No
	CYP2C19 inhibitor	Yes	Yes
	CYP2C9 inhibitor	Yes	Yes
	CYP2D6 inhibitor	No	No
	CYP3A4 inhibitor	Yes	Yes
Excretion	Total clearance (log ml/min/kg)	0.45	0.775
Toxicity	AMES toxicity	No	Yes
	hERG I inhibitor	No	No
	hERG II inhibitor	No	No
	Skin sensitization	No	No

In terms of metabolism, 15K has been found to be a non-substrate and non-inhibitor of CYP2D6 enzyme. A noninhibitor of CYP2D6 means that it wouldn’t interfere in any kind of biotransformation of any compound metabolized by CYP2D6 enzyme. However, it has been predicted to be a substrate and inhibitor of CYP3A4 enzyme demonstrating that it might interfere the metabolism of drugs that are metabolized by this enzyme. Similarly, it may work as the inhibitor of CYP1A2, CYP2C19, and CYP2C9 enzymes. Predicted total clearance of 15K implies moderate level of clearance through kidney, liver, and lungs. This result also indicates a medium level of protein binding capacity of this drug.

Toxicity of 15K was predicted based on the AMES toxicity test, hERG receptor binding, and skin sensitivity test, and the predicted results demonstrate that it can be a non-mutagenic drug without showing toxicity to the heart and irritation to the skin. Surprisingly, most of the predicted ADMET values from pKCSM server comply with the results obtained from ADMETlab 2.0.

In silico pass prediction server was used to predict the highest probable pharmacological properties of 15K and the results show that it can be highly active against hypoxia-inducible factor 1A (Table 8). The other pharmacological properties include anti-inflammatory and analgesic effects. Predicted physicochemical properties support 15K as a good orally bioavailable drug (Table 9) which follows all the druglikeness rules introduced by Lipinski et al.^35^, Ghose et al.^36^, Veber et al.^37^, Egan et al.^38^, and Muegge et al.^39^.

**Table 8:** Pharmacological properties of 15K obtained through in silico PASS prediction, and the properties on which 15K showed more than 70% probability to be active are presented here.

Properties	Pa (%)	Pi (%)
HIF1A expression inhibitor	0.98	0.002
Anti-inflammatory	0.93	0.004
Analgesic	0.86	0.005
Analgesic, non-opioid	0.73	0.005

*HIF1A* Hypoxia-inducible factor 1-alpha, *Pa* Probability to be active, *Pi* Probability to be inactive.

**Table 9:** Predicted physicochemical properties and druglikeness of 15K.

Physicochemical properties	
Molecular weight (g/mol)	442.47
No. of heavy atoms	33
No. of aromatic heavy atoms	22
No. of rotatable bonds	8
No. of H-bond acceptors	6
No. of H-bond donors	0
Molar refractivity	120.02
TPSA^a^ (Å^2^)	88.24
**Druglikeness**	
Lipinski (Pfizer)	Yes; 0 violation
Ghose (Amgen)	Yes
Veber (GSK)	Yes
Egan (Pharmacia)	Yes
Muegge (Bayer)	Yes

^a^Topological polar surface area.

### In vitro toxic effects of 15K

**Figure 10:**
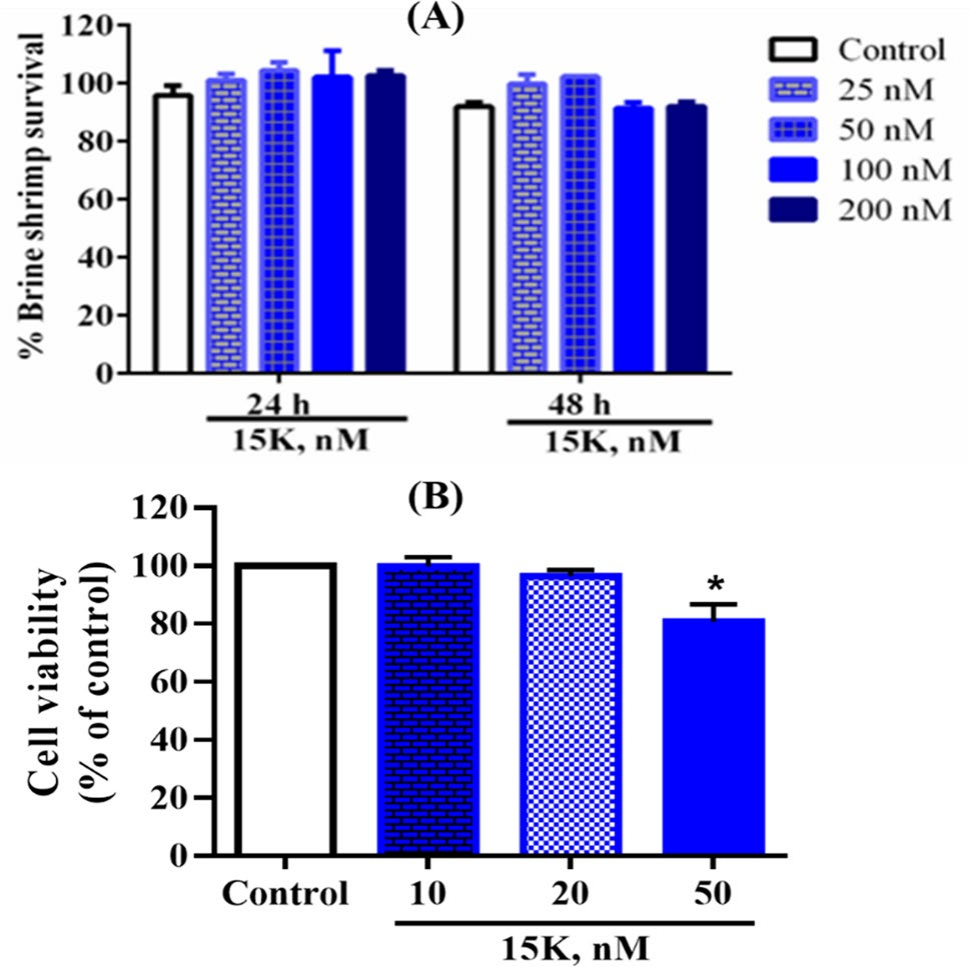
In vitro cytotoxic effects of 15K on brine shrimps (**A**) and 3T3L1 fibroblast cells (**B**) (*p < 0.05, control vs treatment).

Toxicity of 15K was evaluated by using brine shrimp lethality test. 15K was tested using 4 different concentrations and as shown in Fig. 10, it did not exhibit toxicity against brine shrimp at the highest 200 nM concentration after 24 and 48 h treatment. It also did not induce notable toxicity up to 20 nM on 3T3L1 fibroblast cells, while it significantly inhibited cells viability at 50 nM.

### PPI network and enrichment analysis

**Figure 11:**
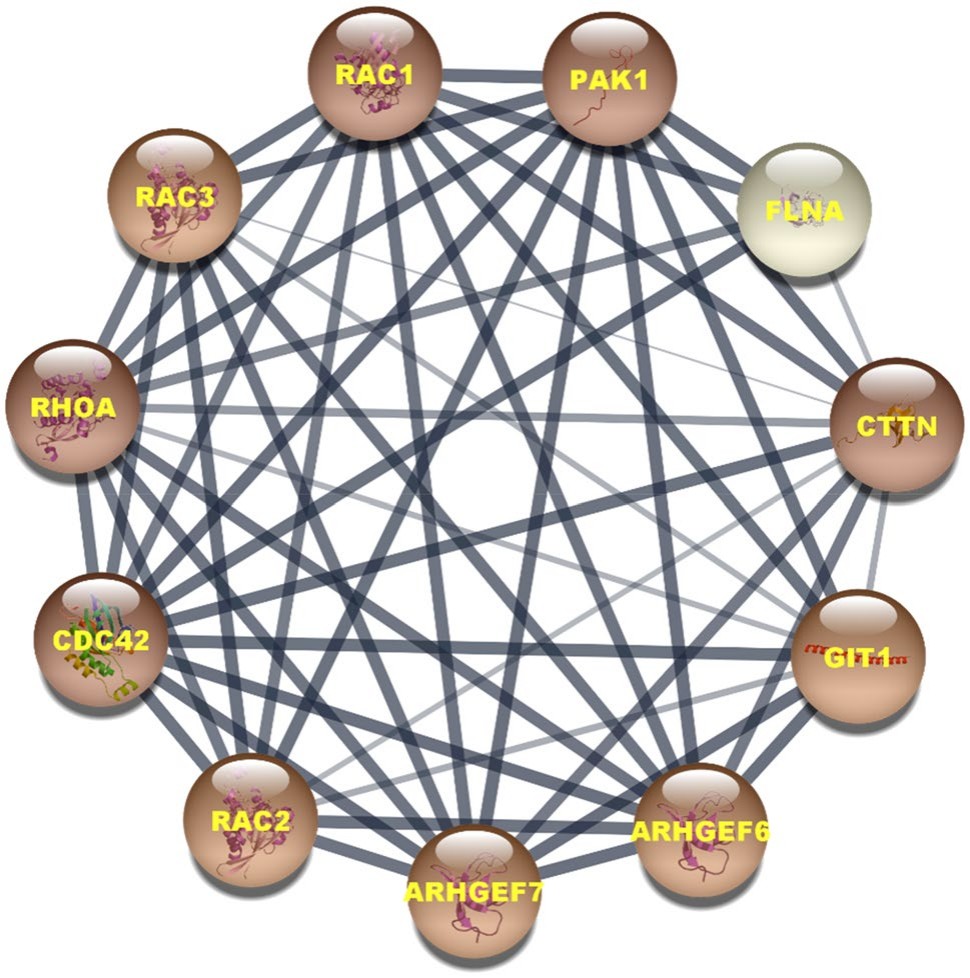
Protein–protein interaction network constructed with PAK1. Nodes with the dark color represents the highest degree value in the network.

The prepared PPI network by PAK1 shows that it consists of 11 nodes and 50 edges (Fig. 11). Five nodes- PAK1, Rac1, Cdc42, Ras homolog family member A (RHOA), and cortactin (CTTN) are significantly involved in this network as proved by their highest number of degree (10). The degree value for all other nodes except for filamin-A (FLNA) is 9. PAK1, Rac1, Cdc42, RHOA, and CTTN also show the highest value for betweenness centrality and stress, while for other proteins both the betweenness centrality and stress are zero. The closeness centrality for that five proteins is also higher than those for other proteins and the lowest closeness centrality is shown by FLNA.

**Figure 12:**
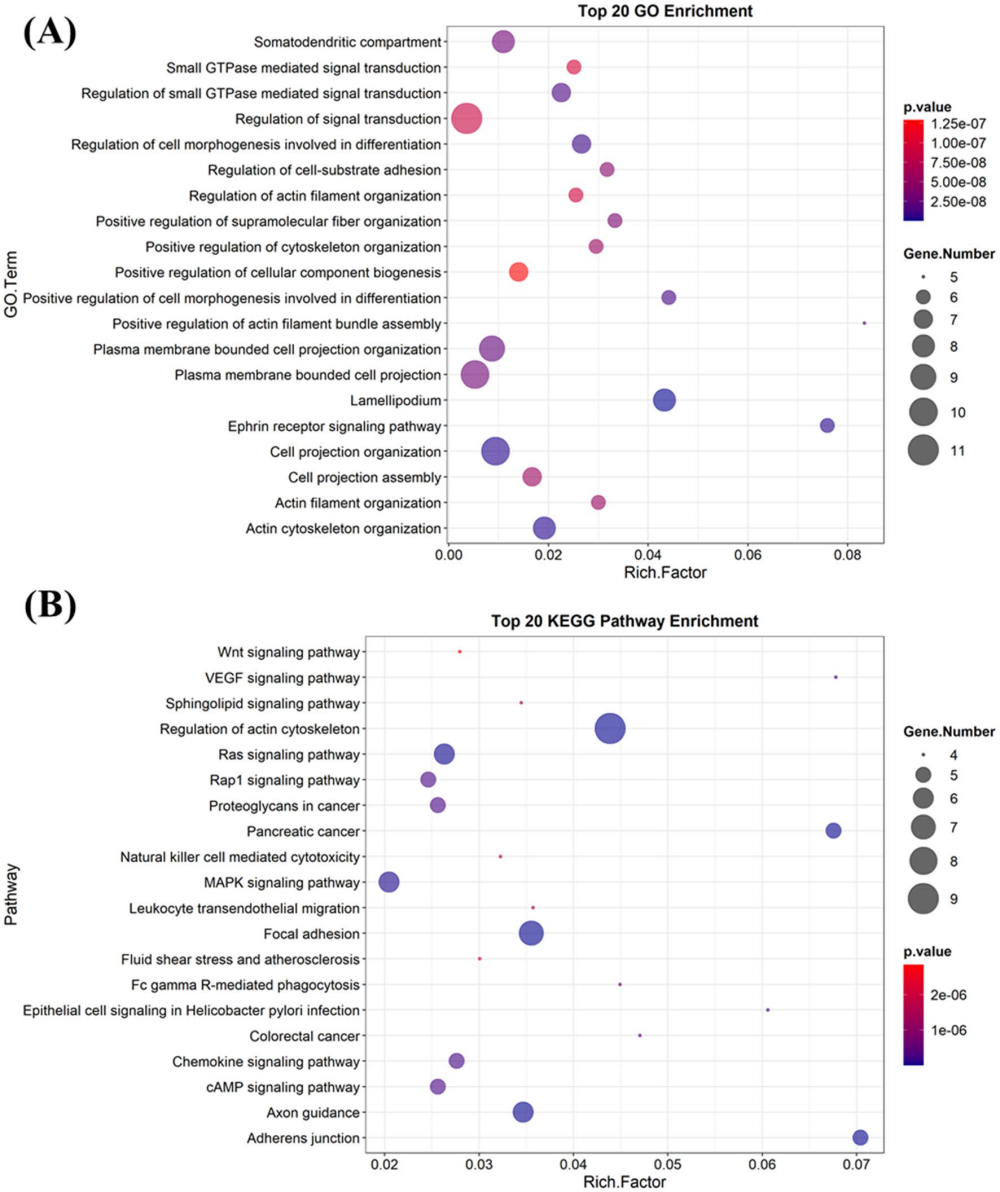
To 20 gene ontology enrichment (**A**) and KEGG pathway enrichment (**B**) analyzed by PAK1 and its other network proteins.

GO enrichment analysis based on 11 targets from the predicted PPI network demonstrates that most of the GO components/processes/functions are relevant to the cellular structure organization and signal transduction regulation. The highly enriched GO terms include actin cytoskeleton organization, cell projection organization, regulation of signal transduction, plasma membrane bounded cell projection, plasma membrane bounded cell projection organization, lamellipodium, etc. (Fig. 12A). All these enriched GO terms can eventually enrich several pathways in cancer proved by the KEGG enrichment analysis prediction (Fig. 12B). The highly enriched pathways are regulation of actin cytoskeleton, Ras signaling pathway, mitogen‑activated protein kinase signaling pathway, focal adhesion, exon guidance, etc.

## Discussion

Protein kinases catalyze phosphorylation reaction and activate a wide array of signaling pathways responsible for cell growth, survival, differentiation, and migration. Small molecule kinase inhibitors therefore attracted attention as promising cancer therapeutics, but most of the developed inhibitors are ATP-competitive that may target other kinases due to having conserved binding features. Thus, cancer drug hunters started looking for non-ATP competitive allosteric inhibitors which have significant advantages over ATP-competitive inhibitors with greater specificity and lower off-target toxicity. In this study, we evaluated 15K, which is a PAK1 inhibitor, using computational techniques and found that it can selectively bind with the allosteric site of PAK1 without targeting other PAK isoforms (PAK2–6) and thereby not showing any toxicity to human body so does pan-PAK inhibitors.

Thermodynamic properties of 15K show that it has greater negative values for electronic energy, enthalpy, and Gibbs free energy than those shown by ketorolac, indicating that 15K has improved thermodynamic features compared with ketorolac^30^. In addition, the higher dipole moment demonstrates high polarity features of 15K than its precursor ketorolac and the tested compound inhibitor 2. Dipole moment is an indicator of drug-receptor interaction, which may facilitate hydrogen bond interactions with the target protein^40^. Molecular orbital properties reveal that the HOMO–LUMO gap is lower in 15K and thus its hardness is low, and softness is high compared to both ketorolac and inhibitor 2. Drugs with higher softness are highly desirable as they can be degraded promptly after showing their pharmacological effects without exerting any unwanted toxicity^41^.

Our group synthesized 15K using “Click Chemistry” technique and proved it to be a more water-soluble and cell permeable azo derivative of ketorolac^9^. Click chemistry technique was introduced by Kolb et al.^42^, based on the principal that coupling of 1,2,3-triazole ring with the carboxylic group in presence of copper catalyzer forms a water-soluble ester. In silico ADMET analysis reveals that 15K can be a poorly water-soluble drug with the log mol/L value of − 4.26. ADMET analysis predicts that it, after ingestion, can be transmitted through intestinal barrier via passive transcellular diffusion. Using multi-drug resistant breast cancer cell line- EMT6, we proved that 15K has the higher cell permeability than ketorolac^9^, which is also justified by the calculated polar surface area (PSA) (88.24 Å^2^). A small molecule drug with less than 100 Å^2^ PSA can show good membrane permeability features^43^. However, it cannot pass the BBB due to having a higher molecular weight than 400 Da^44^; our ADMET analysis also predicted the same. The BBB is a dynamic hindrance ensuring the brain against undesirable substances, and 15K would therefore not show any physiological side effects.

Molecular interaction analysis explores that 15K shows strong binding affinity with PAK1 crystal (binding energy − 8.6 to − 9.2 kcal/mol), forming the highest number of hydrogen bond interactions (three) regardless of the PAK1 crystal structures, while ketorolac showed a varied interaction with different PAK1 crystals. Most importantly it showed two hydrogen bond interactions with the allosteric residue Glu315, similar to inhibitor 2. Several previous reports have already proved that Glu315 is a novel allosteric residue in αC helix, which is responsible for binding the selective PAK1 inhibitors^14, 21^. 15K also can strongly interact with DFG motif residue Asp407 which is another allosteric residue adjacent to the ATP-binding site^14^. During our analysis, 15K did not show notable interaction with other PAK isoforms (PAK2–6). Although the binding affinities with a PAK2 and PAK4 crystal were a bit higher than the normal range, it didn’t show any hydrogen bond interaction either with the active site residues or allosteric residues, which clarifies its weak interaction with PAK2 and PAK4 and thereby demonstrating that 15K has selectivity towards PAK1. When 15K was docked with multiple PAK1 conformers, it exhibited stronger binding affinities than inhibitor 2.

Drug-protein complexes for both 15K and inhibitor 2 were chosen for MD simulation to investigate their stability over the time. Though the average RMSD values of 15K and inhibitor 2 are higher compared to the apo form, they still indicate structural stability by other parameters, such as by the Rgyration and SASA (Fig. 6B,C). RMSF data demonstrate that 15K-PAK1 complex residues show lower fluctuation than other complexes. Importantly, the higher number of hydrogen bonds formation in 15K-PAK1 complex during MD simulation indicates the higher stability of 15K-PAK1 complex over inhibitor 2-PAK1 complex and apo form. The hotspot allosteric residues–Glu315, Met344, and Asp407–show less fluctuation in 15K-PAK1 complex than what they show in case of two other systems. PCA analysis categorizes 15K-PAK1 complex into a distinct group, clearly indicating that 15K-PAK1 complex bears a substantial structural and energy variation favoring its strong interaction with PAK1. MD simulation results firmly support the stable molecular interactions of 15K with PAK1 and therefore it can be considered as a potent allosteric ligand for PAK1 inhibition.

SwissADME prediction reveals that 15K follows all the druglikeness rules according to Lipinski et al.^35^, Ghose et al.^36^, Veber et al.^37^, Egan et al.^38^, and Muegge et al.^39^, and the main features behind its druglikeness are low molecular weight (< 500), less than 5 hydrogen bond donors, and less than 10 hydrogen bond acceptors. Likewise, it would not cause any mutation, toxicity to the heart and skin as the prediction reveals. In vitro toxicity evaluation confirms it to be not toxic to the brine shrimp and fibroblast cells as well. At 50 nM concentration, a 2 × higher concentration than the IC_50_ value against A549 lung cancer cells, it doesn’t cause the death of *C. elegans* rather extending the lifespan of worms^12^. All these experiments together reinforce that 15K could be used as a safe drug, while ketorolac poses hepatotoxicity and renal toxicity^45^. Toxicity of 15K is therefore more specific towards cancer cells through inhibiting PAK1. Network and enrichment analysis also direct that it can breakdown the PAK1-driven protein network and in turn induces potent anticancer effects.

**Figure 13:**
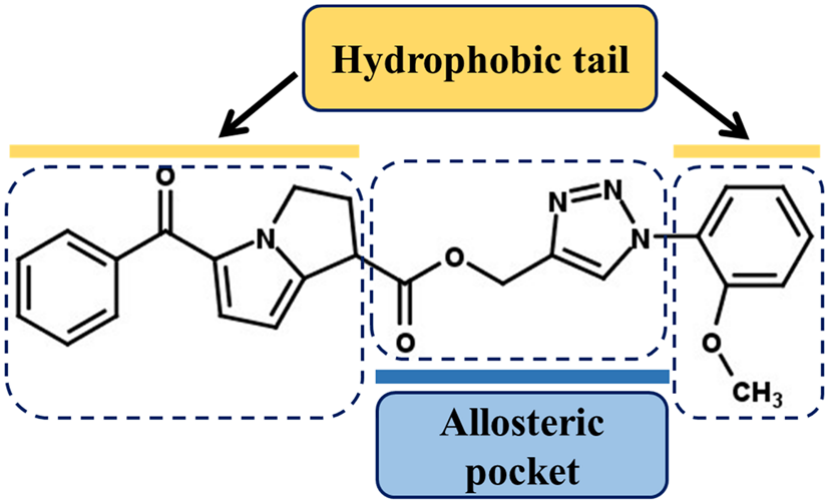
Structure–activity relationship showing the proposed binding orientation of 15K with PAK1 protein domains.

Based on the significant anticancer and anti-PAK1 performance of 15K, we designed and synthesized 1,2,3-triazolyl amide derivative of ketorolac and also synthesized an array of 1,2,3-triazolyl ester derivatives using other NSAIDs having COOH moiety including aspirin, naproxen, ibuprofen, mefenamic acid and indomethacin. But unfortunately, all those derivatives did not show any anticancer effects on A549 lung cancer cells and B16F10 melanoma cells even at high concentration (200 nM) when tested by both trypan blue and MTT assays. Thus, they were not further assessed for anti-PAK1 activities. These results support that the ester bond of 15K has a significant role in its binding with PAK1 and it also has some novel features to be a potent anti-cancer drug. We here propose an SAR of 15K in Fig. 13. 1,2,3-Triazol moiety and the ester bond play a role in identifying and binding with the allosteric pockets, while the terminal ketorolac and methoxy benzene moieties are responsible for establishing hydrophobic bonds with PAK1.

Although this study is limited for not showing any experimental evidence in support of strong binding characteristics of 15K, it warrants co-crystallization studies of 15K with PAK1 to confirm its binding to an allosteric site and to reveal novel interactions. Future studies are also urgent to categorize 15K whether it is type III or type IV allosteric inhibitor.

In conclusion, our computational analysis clearly depicts that 15K can be a highly reactive and safe small molecule drug having all druglikeness features and it can strongly bind with the allosteric site of PAK1. It could act as a selective allosteric PAK1 inhibitor without showing any toxic side effects and can therefore be incorporated in clinical trials for anticancer drug development. Our findings could be used as a guide to study the crystallization of 15K-PAK1 complex to explore the novel interactions of 15K with different PAK1 domains. Besides, this study can be an important guideline in organic synthesis pipeline to develop more potent anticancer drugs, particularly the information that the ester bond and triazole moiety should be kept intact.
